# Error in a Figure

**DOI:** 10.1001/jamanetworkopen.2022.7178

**Published:** 2022-03-18

In the Research Letter titled “Analysis of Ranitidine-Associated *N*-Nitrosodimethylamine Production Under Simulated Physiologic Conditions,”^1^ published January 29, 2021, an error has been corrected in the Figure. In Panel A, the range of sodium nitrite concentrations was truncated and the lower levels (0 to 0.5 mmol/L) were omitted from the line plot. The revised figure indicates that in the absence of exogenous nitrite, a mean of 921 ng of *N*-nitrosodimethylamine was detected from a ranitidine cool mint 150 mg branded tablet. This change does not alter the authors’ conclusions. This article has been corrected.^1^
